# *Mycobacterium africanum*—Review of an Important Cause of Human Tuberculosis in West Africa

**DOI:** 10.1371/journal.pntd.0000744

**Published:** 2010-09-28

**Authors:** Bouke C. de Jong, Martin Antonio, Sebastien Gagneux

**Affiliations:** 1 MRC Laboratories, Bacterial Diseases Programme, Fajara, The Gambia; 2 New York University Division of Infectious Diseases, New York, New York, United States of America; 3 Institute for Tropical Medicine, Mycobacteriology Unit, Antwerp, Belgium; 4 MRC National Institute for Medical Research, London, United Kingdom; 5 Swiss Tropical and Public Health Institute, Basel, Switzerland; Institut Pasteur, France

## Abstract

*Mycobacterium africanum* consists of two phylogenetically distinct lineages within the *Mycobacterium tuberculosis* complex, known as *M. africanum* West African 1 and *M. africanum* West African 2. These lineages are restricted to West Africa, where they cause up to half of human pulmonary tuberculosis. In this review we discuss the definition of *M. africanum*, describe the prevalence and restricted geographical distribution of *M. africanum* West African 1 and 2, review the occurrence of *M. africanum* in animals, and summarize the phenotypic differences described thus far between *M. africanum* and *M. tuberculosis* sensu stricto.

## Introduction

**Figure 1:**
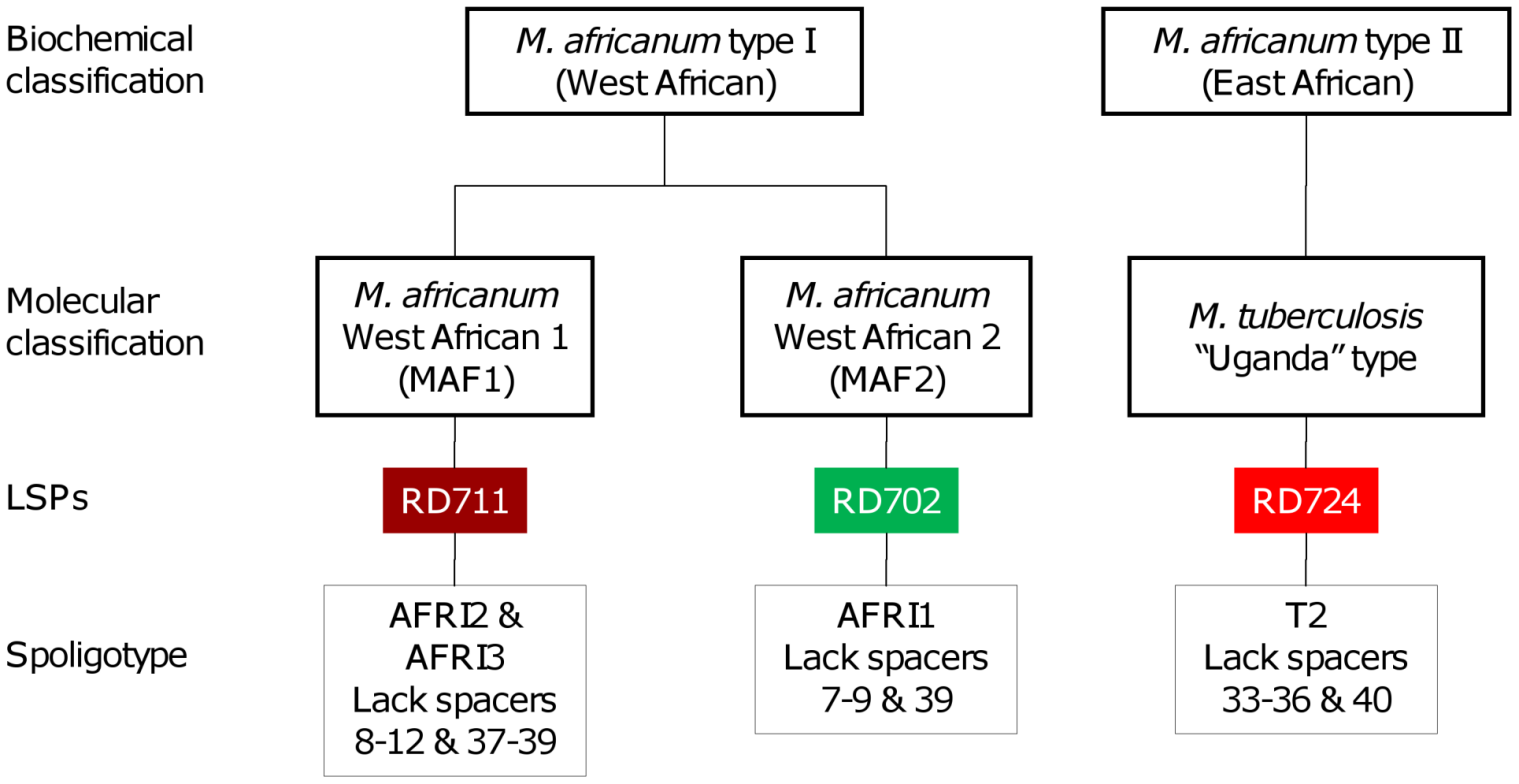
Nomenclature of *M. africanum* as related to its biochemical and molecular classification. Biochemical classification is reviewed in Table 1. LSPs, large sequence polymorphisms [5]. Spoligotype signatures are reviewed in [16].

**Figure 2:**
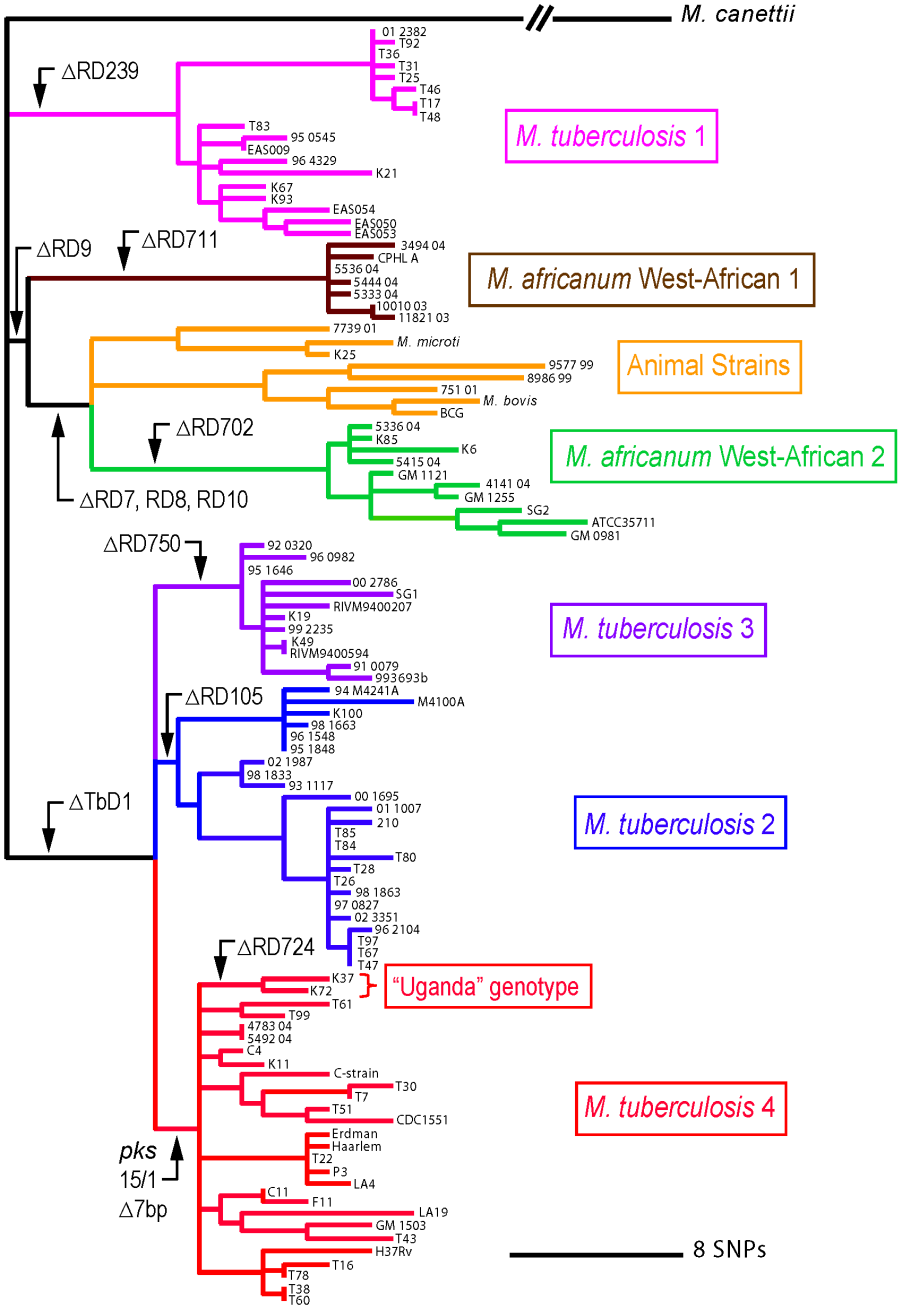
The position of *M. africanum* in the global phylogeny of the *M. tuberculosis* complex (MTBC) as originally published by Hershberg et al.[23]. This phylogeny is based on over 65 kb of DNA sequence data (89 concatenated gene sequences) in each of 108 strains of the MTBC and was inferred using maximum parsimony, which resulted in a single tree with negligable homoplasy [23]. Analysis by the neighbor-joining method resulted in an identical tree topology with high statistical support for all main branches [23]. This phylogeny has been referred to as the most robust and most detailed phylogeny of the MTBC to date, and thus should be considered as the new gold standard for classification of the MTBC [87]. The six main MTBC lineages adapted to humans and the animal strains are indicated in different colors. The human MTBC lineages include four *M. tuberculosis* lineages and the two *M. africanum* type I lineages. The “Uganda” genotype (formally referred to as *M. africanum* type II), which is a sub-lineage within *M. tuberculosis* lineage 4 (also known as the Euro-American lineage), is also shown. These lineages are completely congruent to previous classifications based on LSPs [15], [23], [78]. Black arrows indicate genomic regions or regions of difference (RDs) that are deleted in all descendent strains belonging to a particular lineage or sub-lineage. The scale indicates the genetic distance as number of SNPs (adapted from Figure 1 in [23] with additional data from [5]).

*Mycobacterium africanum* causes up to half of human tuberculosis (TB) in West Africa [1],[2]. It was first described as a distinct sub-species within the *Mycobacterium tuberculosis* complex (MTBC) by Castets and colleagues in 1968 [3]. *M. africanum* yields variable results on classical biochemical characterization, which has complicated the proper classification of the sub-species. This classification has been revised since the advent of molecular genotyping techniques (Figure 1) [4]. Thus, *M. africanum* type I (West African clade) has recently been sub-divided into *M. africanum* type I, West African 1 (MAF1), prevalent around the Gulf of Guinea, and *M. africanum* type I, West African 2 (MAF2), prevalent in western West Africa [5] (Figure 1). The *M. africanum* type II (East African clade) has been reclassified into *M. tuberculosis* sensu stricto (Figure 1) [4], and is indicated as “Uganda” genotype in Figure 2. Castets wrote a French review on *M. africanum* in 1979 [6]. While both *M. africanum* type I and type II were previously reviewed by Onipede and colleagues [7], this review focuses on the two West African lineages within *M. africanum* type I, MAF1 and MAF2. We use the term “*M. africanum*” when referencing studies in which no molecular distinction between MAF1 and MAF2 was included or when MAF1 and MAF2 were described together.

## Methods

We searched PubMed, Web of Knowledge, and Embase databases using the terms “Mycobacterium africanum” and “M. africanum”, and identified further references in these articles.

## Results

### Definition of *M. africanum*

In 1968 in Dakar, Senegal, Castets and colleagues reported mycobacterial strains that, in biochemical testing, were intermediaries between *M. tuberculosis* and *Mycobacterium bovis*, and named these mycobacteria *Mycobacterium africanum* [3]. Like *M. tuberculosis*, *M. africanum* strains were found to be sensitive to pyrazinamide. However, like *M. bovis*, *M. africanum* tended to be nitrate negative, a weak producer of niacin, and to grow microaerophilically in media supplemented with pyruvate, albeit with variable results (Table 1, [8]). Meissner described similar isolates from Ghana in 1969 [9]. On Lowenstein-Jensen solid agar, *M. africanum* grew more slowly than *M. tuberculosis*, with cultures occasionally yielding growth only after 10 weeks, compared to 3–4 weeks in *M. tuberculosis* [10], [11]. Castets thus recommended a 90-day incubation for isolation of *M. africanum* [6]. In minimal media using glucose as sole carbon source, *M. africanum* could not use L-alanine as sole nitrogen source, similar to *M. bovis* [12]. Using colony morphology and biochemical results, a distinction was made between the Dakar variety of *M. africanum*, which likely corresponds to MAF2, and a Yaounde variety, which likely corresponds to MAF1, as well as a Rwanda variety that may have corresponded with the former East African *M. africanum* type II (i.e., today's Uganda genotype of *M. tuberculosis*) [13], [14]. However, studies based on molecular typing have shown that *M. africanum* isolates can exhibit a whole range of biochemical phenotypes [4], which complicates unambiguous classification based on phenotypic testing. By contrast, molecular-based taxonomy is more reliable if appropriate molecular markers are used. Based on the traditional genotyping tools used for routine molecular epidemiological investigation (i.e., IS6110 RFLP and spoligotype analysis), *M. africanum* exhibits a few characteristics that, however, are not always diagnostic [15]. For example, spoligotype patterns tend to permit unambiguous classification, lacking spacers 8 through 12 and 37 through 39 in MAF1 and spacers 7 through 9 and 39 in MAF2 [2], [16].

**Table 1:** Classification of *M. africanum* Relative to *M. tuberculosis* and *M. bovis*.

Characteristic	*M. africanum* Type I		*M. tuberculosis*	*M. bovis*	Reference
	West African 1	West African 2			
**Morphological**					[3]
Colony appearance	Dysgonic	Dysgonic	Eugonic	Dysgonic	
Depth of growth	Microaerophilic	Microaerophilic	Aerophilic	Microaerophilic	
**Biochemical**					[1], [13], [17]
Nitrate reductase	Negative to weakly positive	Negative to weakly positive	Present	Absent	
Niacin production	Negative to weakly positive	Negative to weakly positive	Present	Absent	
**Drug susceptibility**					[3]
Pyrazinamide (PZA)	Sensitive	Sensitive	Sensitive	Resistant	
Thiophene-2-carboxylic acid hydrazide (TCH)	Sensitive	Sensitive	Resistant	Sensitive	
**Molecular**					
Large sequence polymorphisms	RD711	RD702			[4], [5]
SNPs—see Table S1					[23]
Spoligotype analysis	AFRI 2 & 3; absence of spacers 8–12 & 37–39	AFRI 1; absence of spacers 7–9 & 39	Absence of spacer 34	Absence of spacers 39–43	[16]
IS6110 RFLP	Average of 10 bands	Average of 5 bands			[17], [18]

However, for strains that lack the MAF1- or MAF2-specific spacers as well as spacers 33–36, additional molecular tests are required for classification, as such spoligotype patterns could also indicate Euro-American *M. tuberculosis*. Members of MAF1 typically show more than five IS6110 copies and MAF2 generally harbors five or fewer IS6110 copies [17], [18]. The completion of the first *M. tuberculosis* genome sequence facilitated the development of a mycobacterial classification system based on genomic deletions or large sequence polymorphisms (LSPs) [19]. Because the MTBC has a clonal population structure with essentially no horizontal gene exchange, genomic deletions are irreversible and hence represent robust phylogenetic markers. The presence or absence of particular genomic regions of difference can be used to define specific lineages within the MTBC [20]. Following such an approach, initial studies established that MAF1 and MAF2 are distinct lineages, with MAF1 sharing the RD9 deletion with MAF2 and *M. bovis*, while MAF2 and *M. bovis* share additional deletions of RD7, RD8, and RD10 (Figure 2) [21], [22]. These studies also demonstrated that *M. africanum* type II is in fact a member of *M. tuberculosis* sensu stricto as it contains the TbD1 deletion, which is a characteristic marker for a subset of *M. tuberculosis* (Figure 2) [21]. Subsequent studies in which *M. africanum* DNA was compared to *M. tuberculosis* H37Rv using comparative genome hybridization identified additional LSPs that are specific to MAF1 (RD711) and to MAF2 (RD702; Table 1) [4], [5].

Recently, a large-scale multilocus sequence analysis identified multiple single nucleotide polymorphisms (SNPs) specific to MAF1, MAF2, and former *M. africanum* type II (Figure 2, Table S1) [23]. These SNPs can thus be used to define these *M. africanum* lineages unambiguously [15]. The study by Hershberg and colleagues [23] also highlights the large genetic distance that separates MAF1 and MAF2 when compared with other lineages within the MTBC (Figure 2). This high genetic diversity might lead to important phenotypic differences among and between MAF1 and MAF2.

Several other genotyping screens for differentiation of the various species of the MTBC have been developed, such as a PCR protocol in which MAF1 and MAF2 can be distinguished based on LSPs and SNPs [24]. A recent publication identified further polymorphisms specific for MAF1 and for MAF2, as well as two SNPs in *rpoB* that define sub-lineages within MAF2 [25]. However, the current commercial speciation tests do not distinguish between MAF1 and MAF2 (e.g., GenoType MTBC, Hain Lifescience, Germany).

### Prevalence and Distribution of *M. africanum*

**Figure 3:**
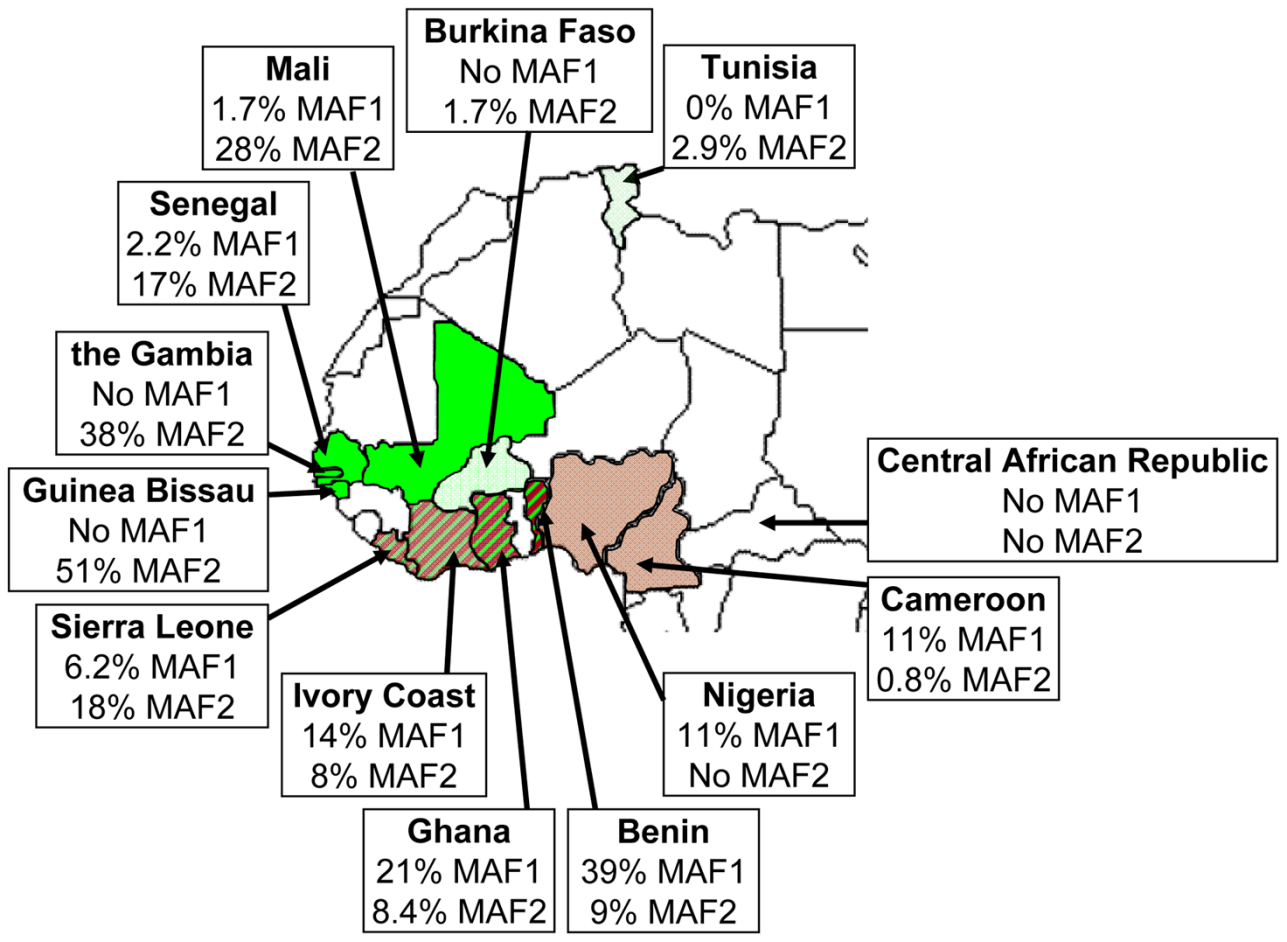
*M. africanum* prevalence in Western African countries. Prevalence figures were derived from the most recently published studies from Table 2 and unpublished data from Tunisia, Senegal, Cote d'Ivoire, Cameroon, and the Central African Republic extracted from the SITVIT2 proprietary database of Institut Pasteur de la Guadeloupe. If studies did not list the prevalence of MAF1 and/or MAF2, the estimates were based on interpretation of the spoligotype patterns. Areas with MAF1 are shaded in brown and areas with MAF2 are shaded in green, with striped shading if prevalence of MAF1 and MAF2 exceeds 5% each. Map courtesy of http://www.theodora.com/maps/, used with permission.

Paleopathological investigation using spoligotype analysis of human remains from Egypt's Middle Kingdom (c. 2000–1600 B.C.) identified MAF2 alongside *M. tuberculosis* [26], [27], although *M. africanum* has not been identified in North or East Africa since [28] and has never been identified in Southern Africa [29]. Studies in Senegal conducted in the 1970s showed that the prevalence of *M. africanum* in that country varied by region and averaged 21% among smear-positive pulmonary TB patients [30] (Table 2). Studies in other West African countries, using biochemical speciation, suggested a prevalence of *M. africanum* ranging from 31% in Mali and Burkina Faso to 66% in Benin [13]. Based on molecular genotyping results, the prevalence of MAF1 appears highest in Benin (39%) and Ghana (21%), with a distribution as shown in Figure 3. Interestingly, the prevalence of MAF1 may be declining in Cameroon; whereas 30 years ago 56% of TB was caused by *M. africanum* based on biochemical speciation [31], an *M. africanum* prevalence of only 9% was reported in a recent study using molecular methods [32]. The prevalence of MAF2 appears highest in Guinea Bissau, where 51% of smear-positive TB was caused by MAF2, based on spoligotyping patterns [1]. As shown in Figure 3, the distributions of MAF1 and MAF2 overlap in southern West Africa, particularly in Ghana [33] and Benin [34].

**Table 2:** Prevalence of *M. africanum* in Different West African Countries.

Country	*n*	West African 1 (MAF1)	West African 2 (MAF2)	*M. africanum* b	Year	Reference
Benin	152			93 (66%)		1982 [13]
Benin	194	55 (39%)	13 (9%)		2005–2006	2009^a^ [34]
Burkina Faso	102			17 (18%)		1979 [52]
Burkina Faso	80			25 (31%)		1982 [13]
Burkina Faso	378			(18%)	1992–1994	1996 [79]
Burkina Faso	120	0	2 (1.7%)			2007^a^ [80]
Cameroon				(56%)		1971 [31]
Cameroon	455	40 (9%)	0		1997–1998	2003 [32]
The Gambia	359	0	138 (38%)		2002–2006	2009 [2]
Ghana	99			42 (42%)		1989 [81]
Ghana	64			13 (20%)	2003	2007 [82]
Ghana	1921	8.4%	21%		2001–2004	2008 [49]
Guinea Bissau	229	0	117 (51%)			1999^a^ [1]
Mali	203			66 (33%)	1973	1974 [11]
Mali	214			66 (31%)		1982 [13]
Mali	119	2 (1.7%)	33 (28%)		2006–2008	2009 [54]
Niger	178			81 (46%)		1982 [13]
Nigeria	55 human; 17 cattle	6 (11%) human; 1 (5.9%) cattle	0			2006 [60]
Senegal	69		7 (10%)		1994–1995	1999^a^ [83]
Sierra Leone	441			122 (28%)	1992–1993	1997 [84]
Sierra Leone	97		17 (18%)		2003–2004	2008 [85]

a Estimates based on interpretation of signature spoligotype patterns in this reference.

b As defined by biochemical criteria.

*M. africanum* has sporadically been identified in areas outside of the West African region, including Germany [9], [35], [36], England [37], California [38], France [39], and Spain [40], both from pulmonary and extra-pulmonary sources. Case reports describe extra-pulmonary TB caused by *M. africanum* as disseminated disease [41], cutaneous disease [42], orchiepididymitis [40], prostatitis [43], pleural disease [44], bone disease [45], brain mass [46], and proctitis [47]. However, in most cases, TB patients carrying *M. africanum* were immigrants from West Africa. The only confirmed outbreak of *M. africanum* outside of West Africa occurred in France, where isolates from the first outbreak of multi-drug-resistant (MDR) TB, diagnosed during the period 1989 to 1992, were initially identified as *M. bovis* on the basis of phenotypic tests. However, later genotypic analysis identified spoligotype patterns consistent with MAF1. The index case in this outbreak originated from Brazil [18], although no MAF1 or MAF2 isolates have been identified in Brazil [48].

Other studies comparing drug resistance have yielded variable results: In Ghana, no difference in drug resistance was identified between *M. africanum* and *M. tuberculosis* [49], whereas a previous regional survey identified more primary resistance among *M. africanum* isolates [13]. The Yaounde variant of *M. africanum* (corresponding to MAF1) may have higher rates of primary resistance to thiacetazone [50]–[52]. This former first-line anti-tuberculous drug has largely been replaced by rifampicin, because of a higher incidence of side effects, particularly in patients with HIV [53]. A recent abstract reported lower drug resistance among MAF2 isolates from Mali relative to *M. tuberculosis* [54].

### Animal Connection of *M. africanum*

The proximity of MAF2 to the animal isolates on the phylogenetic tree of the MTBC (Figure 2) raises the possibility of an animal reservoir for MAF2. Extensive studies in Senegal in the 1970s screened cows, sheep, and pigs, as well as soil samples, but did not identify any non-human reservoir for *M. africanum* infection [30]. Neither *M. bovis* nor other mycobacteria were detected in these animals. Similarly, a recent extensive search for bovine TB in The Gambia, Senegal, Guinea Bissau, and Guinea neither identified tuberculin skin test–positive cows, nor abattoir samples suggestive of TB [55]. An abattoir survey among sheep and goats in The Gambia did not yield any evidence for mycobacterial disease either [56].

*M. africanum* has sporadically been isolated from animals; it has been cultured from monkeys from Central and West Africa with active TB [57], from monkeys in a French veterinary laboratory [58], from four cows from the same dairy farm in Bangladesh that were thought to be infected by a caretaker (MAF2, all with a shared spoligotype pattern, [59]), and twice from a cow in Nigeria (MAF1 [60], *M. africanum* [61]). *M. africanum* can become widely distributed within the tissues of infected animals, and meat or milk may be a route of exposure to *M. africanum* for humans [62]. Efficient human-to-human transmission of MAF2 by aerosol is, however, suggested by the fact that human contacts sleeping in the same bedroom as MAF2 patients have higher rates of tuberculin skin test positivity than contacts sleeping in a different bedroom, with a similar gradient to the one seen with exposure to *M. tuberculosis* [63]. Close interaction between humans and monkeys is limited in West Africa, and it is unclear whether monkeys form a reservoir for *M. africanum* infection, or whether they were themselves infected by humans or a third animal species.

### Experimental Work on *M. africanum*

A study in cows found that *M. africanum*, unlike *M. tuberculosis*, was equally pathogenic as *M. bovis* [62]. Other animal studies have yielded mixed results, possibly due to differences between the two lineages of *M. africanum*. When infecting guinea pigs with *M. africanum* isolates from Dakar (likely MAF2), virulence, as measured by the relative weight of the spleen, was lower for *M. africanum* than for the laboratory strain *M. tuberculosis* H37Rv. The histopathologic lesions observed in the liver resembled those seen after infection with *M. bovis* Bacillus Calmette-Guérin (BCG), with the absence of caseation [64]. Using isolates from Ghana (where MAF1 is more prevalent than MAF2), *M. africanum* was found to be equally virulent as *M. tuberculosis* in guinea pigs, but avirulent in rabbits [9]. Isolates from Central Africa (MAF1 or *M. tuberculosis*) were found to be equally virulent in rabbits compared with *M. tuberculosis* [65]. Tuberculin skin tests, using purified protein derivative (PPD) derived from the respective organisms as well as from *Mycobacterium avium*, showed cross-reaction between *M. tuberculosis* and *M. africanum* PPD both in humans and in animals [66], [67].

In a recent study, BALB/c and C57BL/6 mice were infected with different clinical MAF2 isolates by intravenous tail vein infection and by intra-tracheal aerosol, and bacterial replication in spleen and lungs was compared to infection with *M. tuberculosis* H37Rv (K. Huygen, unpublished data). Significant differences in growth were observed between MAF2 isolates: some isolates displayed the same virulence as *M. tuberculosis* reflected by increasing numbers of CFU in lungs, while other MAF2 isolates showed an attenuated phenotype with little colonization of the lungs. These findings suggest that phenotypic differences exist within the lineage of MAF2 in mouse models, which may in turn explain the variable results in guinea pigs and rabbits.

### Clinical and Epidemiological Differences between *M. africanum* and *M. tuberculosis*

In The Gambia, where we identified *M. africanum* (exclusively MAF2) in sputa of 38% of smear-positive TB patients, we identified various clinical and epidemiological differences between MAF2 and *M. tuberculosis* [68], [69]. In a recent multivariable analysis, we compared patients infected with MAF2 (*n* = 289) relative to the Euro-American lineage of *M. tuberculosis* (EAMTB, *n* = 403), which represent 90% of *M. tuberculosis* sensu stricto isolates in The Gambia [70]. We found that TB patients infected with MAF2 were more likely to be older, to be HIV infected, and to be severely malnourished (Table 3). MAF2 patients were more likely to have more than half the lung fields involved on chest X-ray, despite a similar duration of cough. There was no difference in the proportion of patients with a BCG scar between the two organisms. MAF2 patients were less likely to produce IFNγ in an ESAT-6/CFP-10 ELISPOT assay (OR 0.32, 95% CI 0.18–0.59, *p*<0.0001), although results of tuberculin skin testing were similar [63]. Similarly, MAF2-exposed household contacts were less likely to respond to ESAT-6 in an IFNγ ELISPOT assay than were those exposed to *M. tuberculosis* [63]. Transmission of MAF2 from TB patients to their household contacts occurred at the same rate as that of *M. tuberculosis*, as measured by the tuberculin skin test at baseline and at 3 months, yet household contacts exposed to MAF2 were less likely to develop TB disease in the next 2 years than were contacts exposed to *M. tuberculosis* [71]. The mortality on TB treatment in HIV-negative TB patients was similar between the two organisms, around 3% [69]. The association with HIV infection and the lower progression to disease in HIV-negative patients suggest that MAF2 is somewhat attenuated compared to *M. tuberculosis*. The older age of MAF2 patients may reflect the lower rate of progression to disease. The lower rate of IFNγ responses against ESAT-6 by TB cases infected with MAF2 and their exposed contacts may result from defective ESAT-6 secretion, consistent with preliminary results of ESAT-6 immunoblots of culture filtrates of MAF2 and *M. tuberculosis*. It is unclear whether this ESAT-6 secretion defect results from the fact that the *Rv3879c* gene in the RD1 region is non-functional in MAF2 [63]. In a *Mycobacterium marinum* model, *Rv3879c* was found to be essential for ESAT-6 but not CFP-10 secretion [72]. However, an *M. bovis* BCG recombinant strain with a deletion of *Rv3878* to *Rv3881* efficiently secreted the ESAT-6 and CFP-10 antigens. Similarly, a clinical *M. tuberculosis* isolate lacking *Rv3878* and *Rv3879c* was found to secrete both ESAT-6 and CFP-10 [73], and an *M. bovis* null mutant of the *Rv3879c* homolog was not attenuated for growth in guinea pigs [74]. More work is needed to elucidate the molecular basis of the lower ESAT-6 response in TB patients and their household contacts infected with *M. africanum*.

**Table 3:** Epidemiological, Clinical, and Immunological Differences of *M. africanum* Relative to *M. tuberculosis*.

Characteristic	Country	*M. africanum*		OR(95% CI)	*p*-Value	Reference
		West African 1	West African 2			
Molecular clustering (IS6110 RFLP)	Ghana	Lower		0.38(0.3–0.5)	<0.001	[49]
Progression to disease in first two years after exposure	The Gambia		*Lower*	3.1(1.1–8.7)	0.036	[71]
Prevalence of HIV co-infection	The Gambia		*Higher*	2.13(1.2–3.8)	0.010	[68] [86]
	Ghana	Same as MTB			NS	[49]
IFNγ response to ESAT-6	The Gambia		*Lower*	0.32(0.18–0.59)	0.0001	[63], [86]
Age	The Gambia		*Older*	2.0(1.3–2.9)	0.001	[86]
	Ghana	Same as MTB			NS	[49]
Chest X-ray involvement	The Gambia		*Worse*	1.6(1.1–2.1)	0.005	[86]
	Ghana	Less lower lobe disease in HIV-negative patients		0.34(0.2–0.6)	<0.001	[49]

Underlined text indicates results from the study from Ghana, which combined the West African 1 and 2 data.

Italic text indicates results from the study from The Gambia, which only identified West African 2.

CI, confidence interval; ESAT-6, early secreted antigen- 6 kDa; IS6110 RFLP, insertion sequence 6110 restriction fragment length polymorphisms; MTB, *M. tuberculosis*.

Studying nearly 2,000 TB patients from Ghana, 29% of whom were infected with *M. africanum* (MAF1 and MAF2 combined) as opposed to *M. tuberculosis*, no differences in the rates of HIV co-infection, nor chest X-ray severity, were seen between these organisms [49]. HIV-negative *M. tuberculosis* patients were significantly more likely to have exclusively lower zone disease on chest X-ray. Chest X-rays did not differ between MAF1 and MAF2. *M. africanum* patients were significantly less likely to have recently transmitted disease (i.e., be part of a transmission cluster) as opposed to reactivation disease. A lower clustering rate in *M. africanum* compared to *M. tuberculosis* is consistent with the lower progression to disease observed in The Gambia.

Similar to early findings in Dakar [6], we observed a longer time to growth in culture of MAF2 relative to *M. tuberculosis* on primary isolation from sputum, both in liquid and solid medium, adjusted for the degree of smear positivity and extent of disease on chest X-ray (*p*<0.0001, B. de Jong, unpublished data).

### Unanswered Questions

The reason why *M. africanum* has not established itself outside of the West Africa region remains enigmatic. Potential explanations for the limited geographic spread of *M. africanum* despite massive migrations of West Africans to the Americas at the time of the slave trade include the possibility that diseased slaves did not survive the crossing of the Atlantic, or that *M. africanum* did initially establish itself in the New World, but was subsequently outcompeted by *M. tuberculosis*, possibly due to its lower rate of progression to disease relative to *M. tuberculosis*. Moreover, *M. africanum* may not have established itself in the indigenous American people and in their European colonizers if this organism has a host preference for ethnically West African persons. Indeed, a recent study described a host polymorphism found in Ghana that is associated with protection against Euro-American *M. tuberculosis*, yet not against *M. africanum*, and may thus have provided a selective advantage for *M. africanum* in West African populations [75]. Studies of innate immune responses in individuals of different ethnic origins could test the hypothesis that *M. africanum* has adapted to preferentially establishing infection in West African hosts [5].

A West African animal reservoir of MAF2 that sustains a large reservoir of latent infection in humans, and maintains MAF2 in the human population despite a lower rate of progression, would be in line with the phylogenetic relatedness of MAF2 and the animal strains within the MTBC. A larger reservoir of latent infection of MAF2 in The Gambia could in turn explain the association with HIV infection seen in that country, yet not in Ghana. However, a putative animal reservoir is more difficult to invoke for MAF1 given that this lineage is phylogenetically more distant from the classical animal-adapted sub-species of the MTBC (Figure 2).

As DNA extraction from organisms causing latent infection is impossible with the currently available technologies, we need new tools to distinguish between latent infections with different sub-species within the MTBC. We found that antigens from the TbD1 region, present in *M. africanum* but absent from *M. tuberculosis*, failed to induce a T cell response that could be measured in an IFNγ ELISPOT [76]. Availability of the whole genome sequence of a strain of MAF2 [77] will facilitate identification of other potentially differential T cell and B cell epitopes. Establishing such tools would allow us to compare the size of the reservoir of latent infection with *M. africanum* relative to *M. tuberculosis*, and to answer whether MAF2, with its lower rate of progression to disease, protects against progression to disease of *M. tuberculosis*. Such a protection could explain why MAF2, with its lower rate of progression and shared host, has not been out-competed by *M. tuberculosis*.

Understanding the genotypic differences underlying the phenotypic differences between MAF2 and *M. tuberculosis* can greatly enhance our understanding of TB pathogenesis. To this end, the completion of the MAF2 whole genome sequence will facilitate experimental studies on the biological differences between MAF2 and *M. tuberculosis*, such as candidate genes that may underlie the attenuated ESAT-6 response induced by MAF2. In addition, more research should be dedicated to MAF1, as little is known on putative phenotypic characteristics of this *M. africanum* lineage. To successfully address all of these research questions, a comprehensive and multidisciplinary “systems epidemiology” approach will be necessary [78].

In conclusion, MAF 1 and MAF 2 are phylogenetically “ancient” sub-species within the MTBC that show phenotypic differences relative to *M. tuberculosis* (as observed for MAF2 in The Gambia), and may be gradually outcompeted by *M. tuberculosis* (as suggested for MAF1 in Cameroon). Studies on MAF2 can inform on TB pathogenesis, as MAF2, if defective ESAT-6 secretion is confirmed, could provide a model for the effects of ESAT-6 in the natural human host. Conversely, the lower progression to disease of MAF2 makes this organism a potential model for the study of containment of TB. Lastly, the new generations of TB diagnostics and vaccines currently under development need to ensure efficacy in *M. africanum*–endemic areas.

**Learning Points**

- *M. africanum* is a sub-species of the *M. tuberculosis* complex that consists of two distinct lineages, West African 1 and 2
- Unambiguous identification of *M. africanum* requires molecular methods
- *M. africanum* is found only in West Africa, where it causes up to half of human TB
- *M. africanum* West African 2 has distinct phenotypes compared to *M. tuberculosis*, such as a lower progression to disease in exposed contacts, despite a similar rate of transmission
- Infection with *M. africanum* responds to regular TB treatment

**Key Papers**

- Castets M, Boisvert H, Grumbach F, Brunel M, Rist N (1968) [Tuberculosis bacilli of the African type: preliminary note]. Rev Tuberc Pneumol (Paris) 32: 179–184.
- Mostowy S, Onipede A, Gagneux S, Niemann S, Kremer K, et al. (2004) Genomic analysis distinguishes *Mycobacterium africanum*. J Clin Microbiol 42: 3594–3599.
- de Jong BC, Hill PC, Brookes RH, Gagneux S, Jeffries DJ, et al. (2006) Mycobacterium africanum elicits an attenuated T cell response to early secreted antigenic target, 6 kDa, in patients with tuberculosis and their household contacts. J Infect Dis 193: 1279–1286.
- Meyer CG, Scarisbrick G, Niemann S, Browne EN, Chinbuah MA, et al. (2008) Pulmonary tuberculosis: virulence of *Mycobacterium africanum* and relevance in HIV co-infection. Tuberculosis (Edinb) 88: 482–489.
- de Jong BC, Hill PC, Aiken A, Awine T, Antonio M, et al. (2008) Progression to active tuberculosis, but not transmission, varies by *Mycobacterium tuberculosis* lineage in The Gambia. J Infect Dis 198: 1037–1043.
- Hershberg R, Lipatov M, Small PM, Sheffer H, Niemann S, et al. (2008) High functional diversity in *Mycobacterium tuberculosis* driven by genetic drift and human demography. PLoS Biol 6: e311. doi:10.1371/journal.pbio.0060311.

## Supporting Information

Table S1 Single nucleotide polymorphisms (SNPs) that define the various *M. africanum* lineages, based on [23] and [15].
(0.07 MB DOC)
