# Supplementary material for: Mycobacterium africanum—Review of an Important Cause of Human Tuberculosis in West Africa
Source: PLoS Negl Trop Dis. 2010 Sep 28;4(9):e744. doi: 10.1371/journal.pntd.0000744 (PMC2946903; doi:10.1371/journal.pntd.0000744)
Supplement: Table S1 — Single nucleotide polymorphisms (SNPs) that define the various M. africanum lineages, based on [23] and [15]. (0.07 MB DOC) [file pntd.0000744.s001.doc]

| **Table S1.** Single nucleotide polymorphisms (SNPs) that define the various *M. africanum* lineages, based on [23] and [15]. | | | | | | | | |  |  |  |
| --- | --- | --- | --- | --- | --- | --- | --- | --- | --- | --- | --- |
| Lineage | SNP Name | Rv Number | SNP Type | Position | Wild type (H37Rv) Base Pair | Mutant Bas Pair | Codon | Wild Type (H37Rv)Amino Acid | Mutant Amino Acid | LSP | Spoligo Lineage |
| *M. africanum* West African 1 (MAF1) | Rv0006_2265s | 0006 | S | 2265 | taC | taT | 0755 | Tyr | Tyr | RD711 | AFRI2 |
|  | Rv0410c_1993n | 0410c | N | 1993 | Gcc | Acc | 0665 | Ala | Thr |  |  |
|  | Rv0413_0511n | 0413 | N | 0511 | Gtg | Ttg | 0171 | Val | Leu |  |  |
|  | Rv0467_1224s | 0467 | S | 1224 | acC | acT | 0408 | Thr | Thr |  |  |
|  | Rv1980c_0128n | 1980c | N | 0128 | aTt | aAt | 0043 | Ile | Asn |  |  |
|  | Rv2030c_0061n | 2030c | N | 0061 | Cgc | Agc | 0021 | Arg | Ser |  |  |
|  | Rv2450c_0049s | 2450c | S | 0049 | Ttg | Ctg | 0017 | Leu | Leu |  |  |
|  | Rv2628_0286n | 2628 | N | 0286 | Gcg | Acg | 0096 | Ala | Thr |  |  |
|  | Rv2687c_0322n | 2687c | N | 0322 | Gtg | Ttg | 0108 | Val | Leu |  |  |
|  | Rv2949c_0092n | 2949c | N | 0092 | gTt | gGt | 0031 | Val | Gly |  |  |
|  | Rv2957_0447n | 2957 | N | 0447 | ttC | ttA | 0149 | Phe | Leu |  |  |
|  | Rv3132c_0019n | 3132c | N | 0019 | Gtc | Atc | 0007 | Val | Ile |  |  |
|  | Rv3547_0144s | 3547 | S | 0144 | ctG | ctT | 0048 | Leu | Leu |  |  |
|  | Rv3547_0337n | 3547 | N | 0337 | Gac | Aac | 0113 | Asp | Asn |  |  |
| *M. africanum* West African 2 (MAF2) | Rv0288_0211n | 0288 | N | 0211 | Gcg | Tcg | 0071 | Ala | Ser | RD702 | AFRI1 |
|  | Rv0407_0886n | 0407 | N | 0886 | Aag | Ggg | 0296 | Lys | Glu |  |  |
|  | Rv0410c_2207n | 0410c | N | 2207 | aCg | aAg | 0736 | Thr | Lys |  |  |
|  | Rv0429c_0231s | 0429c | S | 0231 | cgC | cgA | 0077 | Arg | Arg |  |  |
|  | Rv1305_0207s | 1305 | S | 0207 | gcG | gcT | 0069 | Ala | Ala |  |  |
|  | Rv2030c_1569s | 2030c | S | 1569 | caT | caC | 0523 | His | His |  |  |
|  | Rv2185c_0351s | 2185c | S | 0351 | ccC | ccA | 0117 | Pro | Pro |  |  |
|  | Rv2744c_0689n | 2744c | N | 0689 | cGg | cAg | 0230 | Arg | Gln |  |  |
|  | Rv2744c_0755n | 2744c | N | 0755 | cCc | cTc | 0252 | Pro | Leu |  |  |
|  | Rv2875_0510s | 2875 | S | 0510 | gtC | gtT | 0170 | Val | Val |  |  |
|  | Rv3589_0710n | 3589 | N | 0710 | gCc | gAc | 0237 | Ala | Asp |  |  |
| "Uganda" genotype | Rv0006_0238n | 0006 | N | 0238 | Acc | Gcc | 0080 | Thr | Ala | RD724 | T2 |
|  | Rv0040c_0619n | 0040c | N | 0619 | Gac | Aac | 0207 | Asp | Asn |  |  |
|  | Rv2949c_0375s | 2949c | S | 0375 | ttC | ttT | 0125 | Phe | Phe |  |  |
